# Promoting Health Literacy in the Workplace Among Civil Servants: Cross-Sectional Study

**DOI:** 10.2196/58942

**Published:** 2024-08-15

**Authors:** Florence Carrouel, Benjamin du Sartz de Vigneulles, Céline Clément, Virginie-Eve Lvovschi, Elise Verot, Valeria Tantardini, Michel Lamure, Denis Bourgeois, Romain Lan, Claude Dussart

**Affiliations:** 1 Health Systemic Process Laboratory (P2S), UR4129, University Claude Bernard Lyon 1, University of Lyon, Lyon, France; 2 Interpsy Laboratory (INSERM UR4432), University of Lorraine, Nancy, France; 3 Research on Healthcare Performance Laboratory (INSERM U1290), University Claude Bernard Lyon 1, University of Lyon, Lyon, France; 4 Hospices Civils of Lyon, Lyon, France; 5 PRESAGE Institute, University Jean Monnet, Saint-Etienne, France; 6 Centre Hospitalier Universitaire de Saint-Étienne (CIC 1408 INSERM), Saint-Étienne, France; 7 Geriatric Rehabilitation and Follow-up Care Service, Centre Hospitalier Universitaire of Rouen, Oissel site, Rouen, France; 8 Anthropologie bio-culturelle, droit, éthique et santé Laboratory (ADES, UMR7268), Centre National de la Recherche Scientifique, Etablissement Français du Sang, Aix Marseille University, Marseille, France

**Keywords:** health literacy, oral health literacy, workplace, civil servant, health promotion, prevention

## Abstract

**Background:**

In 2022, the World Health Organization highlighted the alarming state of oral health (OH) worldwide and urged action to include OH in initiatives on noncommunicable diseases. The population needs improved OH skills and attitudes and an adequate level of OH literacy (OHL) and general health literacy (HL). The implementation of health promotion actions in the workplace, which is a part of most people’s lives, appears to be an opportunity. In France, civil servants have several socioprofessional levels and represent an excellent model with results transposable to the population.

**Objective:**

This study aimed at determining the OHL and HL level of civil servants in France in order to implement specific prevention actions in their workplaces.

**Methods:**

A cross-sectional study of French civil servants was conducted in France from October 2023 to February 2024. Participants completed three validated questionnaires in French: (1) a questionnaire on OH knowledge, (2) the Oral Health Literacy Instrument, French version (OHLI-F; this is composed of reading comprehension and numeracy sections) to assess the OHL level, and (3) the Short Test of Functional Health Literacy in Adults, French version (s-TOFHLA-F) to assess the HL level. The scores for OH knowledge, the OHLI-F, and the s-TOFHLA-F were reported as means (SD) and the 95% CI. These scores were classified into 3 categories: adequate (75-100), marginal (60-74) and inadequate (0-59). ANOVA and binary logistic regression were performed. The OHLI-F reading comprehension and OHLI-F numeracy scores were compared using the Welch 2-sample *t* test and a paired *t* test (both 2-tailed). For the correlation matrix, the Pearson correlation and related tests were computed.

**Results:**

A total of 1917 persons completed the 3 questionnaires, with adequate levels of OHL (n=1610, 84%), OH knowledge (n=1736, 90.6%), and HL (n=1915, 99.9%). The scores on the s-TOFHLA-F (mean 98.2, SD 2.8) were higher than the OHLI-F (mean 80.9, SD 7.9) and OH knowledge (mean 87.6, SD 10.5). The OHLI-F was highly correlated with OH knowledge (*P<*.001), but the OHLI-F and OH knowledge had a low correlation with s-TOFHLA-F (*P*=.43). The OHLI-F reading comprehension score was significantly higher than the OHLI-F numeracy score (*P*<.001). Age, education level, and professional category impacted the 3 scores (*P*<.001). The professional category was a determinant of adequate OHLI-F and OH knowledge scores.

**Conclusions:**

Some French civil servants had inadequate or marginal levels of OH knowledge (n=181, 9.5%) and OHL (n=307, 16%) but none had an inadequate level of HL. Results highlighted the relevance of implementing OH promotion programs in the workplace. They should be nonstandardized, adapted to the literacy level of professional categories of workers, and focused on numeracy skills. Thus, appropriate preventive communication and improved literacy levels are the means to achieve greater disease equity and combat the burden of noncommunicable diseases.

## Introduction

In 2022, the World Health Organization (WHO) highlighted the alarming state of oral health (OH) worldwide and urged action to include OH in initiatives on noncommunicable diseases (NCDs) and universal health coverage [1]. Around 50% of the world’s population (3.5 billion people) has oral disease. The global burden of oral disease exceeds the combined global burden of the 5 most prevalent NCDs by almost 1 billion cases [2].

Oral diseases are key risk factors for NCDs [3] and are caused by a range of risk factors common to many NCDs, including sugar consumption, tobacco use, alcohol use, and poor hygiene, and their underlying social and commercial determinants [1]. Therefore, it is necessary to implement higher standards of oral hygiene and behavior. This goal could be achieved by teaching, educating, and motivating people to follow oral hygiene instructions and by improving their skills and attitudes toward their OH [4]. People must be active and responsible for their own health, and compliance is crucial [5]. One of the determinants of achieving this seems to be the acquisition, for each person, of a sufficient level of health literacy (HL), that is, “the knowledge, motivation and competences to access, understand, appraise and apply health information in order to make judgments and take decisions in everyday life concerning health care, disease prevention and health promotion to maintain or improve quality of life throughout the course of life” [6]. Improving OH literacy (OHL), as demonstrated for HL, remains essential for both improving OH and decreasing OH inequalities [7]. Disparities in OH have been attributed to social, psychological, and structural factors [8]. Literacy can be seen as a complex phenomenon in which a wide range of useful skills can fluctuate according to the cultural context [9-11]. Among factors impacting on literacy levels are personal determinants [6,12], including education [13,14], profession, employment, income, and socioeconomic status [15,16]. There are also societal and environmental determinants, which are likely to include work [17,18].

As work is a key social determinant of health [19], the workplace represents a fundamental context in terms of health. However, in the workplace, OHL and general HL levels remains poorly documented. Better knowledge of these will permit implementing specific health promotion programs in the workplace. Indeed, the workplace represents an opportunity because most adults have a job, and full-time workers spend most of their time in the workplace; work is an important part of most people’s lives [20,21]. Thus, the workplace must play an essential role in prevention [22], and prevention campaigns should be adapted to the specificities of workers [23].

To better understand the link between work, OHL, and HL, we need to characterize them further, particularly through socioprofessional characteristics. The French public service employed 5.67 million civil servants on December 31, 2021, representing almost 1 in 5 jobs. It is divided into 3 parts: the state civil service (essentially administrative activities; 44% of public employment), the territorial civil service (including local-authority employers; 34% of public employment), and the hospital civil service (21% of public employment) [24]. The French public service is particularly interesting to study for a number of reasons. First, these public agents work in a specific context with a guaranteed job and a relatively high degree of stability in their working environment. Second, there are representatives from several socioprofessional categories [25]. Third, there are few occupational physicians in this population, and some organizations have to address this gap in the preventive network. Fourth, the management of health care costs for public service workers is unique in that it is delegated to the mutual system and continues after retirement. As a result, mutual insurance organizations play a major role in preventive activities on behalf of the public authorities, as stated in the latest Occupational Health Plan for the Civil Service [26].

Thus, identifying the level of OHL and general HL in this specific category of workers could help better target and facilitate the implementation of prevention and awareness programs in the workplace. In addition, any differences found according to socioprofessional levels would enable these results to be interpreted regarding the general population.

This study aimed at determining the OHL and general HL level of civil servants in France in order to implement specific prevention actions in their workplace.

## Methods

### Study Design and Setting

This cross-sectional study was conducted in France from October 2023 to February 2024. This study was performed in accordance with the Strengthening the Reporting of Observational Studies in Epidemiology (STROBE) [27] checklist (Table S1 in Multimedia Appendix 1).

### Participants

#### Description

The target population was French civil servants from state services (all professional categories combined, active or retired) who had a digital personal space on the health insurance government website in October 2023 and agreed to receive information at their email address. In the public service, there is a classification into 3 main professional categories: A, B, and C. Each category covers jobs with different responsibilities, qualifications, remuneration, and recruitment conditions. Category A covers hierarchically superior conceptual, managerial, and supervisory grades and positions, as well as teaching positions. Category B includes middle management and application and editorial positions. Category C includes executive positions. The conditions of access to these categories vary according to the level of qualification and graduation. The basic remuneration is different between these categories [24].

#### Inclusion and Exclusion Criteria

To be included in this study, the participants had to be (1) aged at least 18 years, (2) public service agents or former public service agents in 1 of the 3 categories (A, B, or C,), (3) fluent in the French language, and (4) able to understand, accept, and convey in writing their agreement to participate in the study.

Participants were excluded if they (1) had an intellectual disability, (2) had visual acuity problems that prevented them from reading the questionnaire, and (3) were unable to answer any question or finish the questionnaires.

#### Sample Size

The sample size was calculated based on the results of the studies of Sabbahi et al [28], Ramlay et al [29] and Clément et al [30]. The 2-mean formula was applied, with α=.05, power=80%, SD=18, expected difference=10, and expected dropout rate=20%, giving n=51 per group for each professional category of French civil servant (A, B, and C); thus*,* the total sample size required was 153 participants. This sample was in accordance with Cochrane recommendations and the sample sizes of previous studies [15,31,32].

### Study Process

An email invitation was sent out to 348,715 civil servants with a detailed information letter about the study. Persons who agreed to participate and who met the inclusion criteria were included. For people who agreed to participate, a questionnaire was used to check the inclusion and exclusion criteria. Participants completed a questionnaire about their sociodemographic characteristics (eg, age, sex, education level, professional category, and frequency of dental visits), for which the answer to each question was not compulsory in order to respect people’s privacy. Then, they completed 3 questionnaires (in French): the OH knowledge questionnaire [30], the Oral Health Literacy Instrument (OHLI) [30] and the Short Test of Functional Health Literacy in Adults (s-TOFHLA) [33].

### Outcomes

The main outcome was to determine the level of OHL of civil servants in France. The secondary outcomes were to determine the level of OH knowledge; to determine the level of general HL; to identify the determinants of an adequate level of OHL, OH knowledge, and HL; and to assess the correlation between the level of OHL and the level of HL.

### Data Collection (Questionnaire Design)

#### French Version of the OHLI

The French OHLI (OHLI-F) [30] is based on the English OHLI [28]. The OHLI-F is composed of 2 parts. The first part evaluates the capacity to both read and understand information on oral diseases (reading comprehension). The second part evaluates the capacity to understand instructions requiring simple mathematical calculations (numeracy).

The first part has 2 sections (dental caries and periodontal diseases). The dental caries section contains 13 sentences to complete (18 missing words out of 264 words). The periodontal diseases section contains 14 sentences (20 missing words out of 228 words). Four words are given as options for each missing word but only 1 answer is correct. A correct answer is scored 1 point, while an incorrect answer or no answer is scored zero points. This section, which assesses reading comprehension, is self-administered. The second part is a set of printed questions relating to prescriptions for 5 drugs frequently prescribed by dentists, a dental appointment card, and a postextraction procedure sheet. This section contains 19 questions and evaluates numeracy. A correct answer is scored 1 point, while an incorrect answer or no answer is scored zero points. The final score for each part is the sum of the points obtained in that part. The final score for each part (out of 50) is obtained by multiplying the total score for the reading comprehension part by 1.316 (50/38) and the total score for the arithmetic part by 2.362 (50/19). The sum of these 2 scores is the OHLI score, which varies between 0 and 100. The higher the OHLI score, the greater the functional competence in terms of OH. Three levels of OH literacy can be defined using the OHLI score: adequate (75-100), marginal (60-74), and inadequate (0-59),

#### French OH Knowledge Questionnaire

To assess participants’ level of general dental knowledge, an OH knowledge test was used [30]. This test, composed of 7 images, analyzes a broad spectrum of dental topics relating to anatomical structures and physiological processes, dental materials, dental prostheses, treatments, and preventive practices. For each image, the participant associates a word with one of the elements in the image, such as perioral and intraoral structures, oral diseases and conditions, dental obturations, dental prostheses, and various oral hygiene aids. There are 17 elements to recognize. A correct answer to 1 item is scored 1 point, while an incorrect answer or no answer is scored zero points. To obtain a final score out of 100, these points are added together and the sum is multiplied by 5.88 (100/17). Three levels of OH knowledge can be defined using this final score: inadequate (0-59), marginal (60-74), and adequate (75-100).

#### French Version of the s-TOFHLA Questionnaire

The French version of the s-TOFHLA [33] was used. This test assesses written comprehension of health literacy. It consists of 2 sections with 36 missing words. The first section deals with a healthy diet and contains 8 sentences with 16 missing words. The second section deals with a healthy lifestyle and contains 11 sentences with 20 missing words. For each missing word, the participant must choose the correct answer from 4 options. A correct answer to 1 item is scored 1 point, while an incorrect answer or no answer is scored zero points. To obtain a final score out of 100, these points are added together and the sum is multiplied by 2.778 (100/36). Three levels of health literacy can be defined: inadequate (0-59), marginal (60-74), and adequate (75-100).

### Statistical Analysis

The education level was categorized as level 1, or low (<baccalaureate); level 2, or moderate (from baccalaureate to baccalaureate plus 2 years); and level 3, or high (>baccalaureate plus 2 years). Data were analyzed using R (version 3.6.0; R Foundation for Statistical Computing). Sociodemographic numerical and categorical variables were calculated and reported as number (percentage) and mean (SD), respectively. The scores for OH knowledge, OHLI-F, and s-TOFHLA were calculated and reported as mean (SD) and 95% CIs. The influence of the variables (eg, age, gender professional category, and professional situation) on the OHLI scores (OHLI-F, OH knowledge, and s-TOFHLA) was tested using a classic ANOVA. This analysis was supplemented by logistic regressions where the variables to be explained were binarized scores using a threshold equal to 75 (1 if the score is greater than or equal to 75, 0 otherwise). The R packages *gtsummary* and *ggstats* were used in these analyses.

The comparison between the OHLI-F reading comprehension and numeracy scores was performed using the Welch 2-sample *t* test and a paired *t* test (both 2-tailed).

Concerning the correlation matrix between scores, the Pearson correlation parameter was computed and tested for nullity via a usual *t* test. The correlation was assumed low or null for scores between 0.00 and 0.25, low for scores between 0.26 and 0.49, moderate for scores between 0.50 and 0.69, high for scores between 0.70 and 0.89, and very high for scores between 0.90 and 1.00 [34].

### Ethical Considerations

The study was conducted in accordance with the Declaration of Helsinki and approved by the Research Ethics Committee of the University Hospital Centre of Rouen (E2023-4; October 15, 2023). The platform Claroline connect from the University Claude Bernard Lyon 1 used for the online questionnaire was in accordance with the Regulation EU 2016/679 of the European Parliament and the Council of 27 April 2016 on the protection of individuals with regard to the processing of personal data and on the free movement of such data.

## Results

### Sociodemographic Characteristics of the Participants

**Figure 1.**
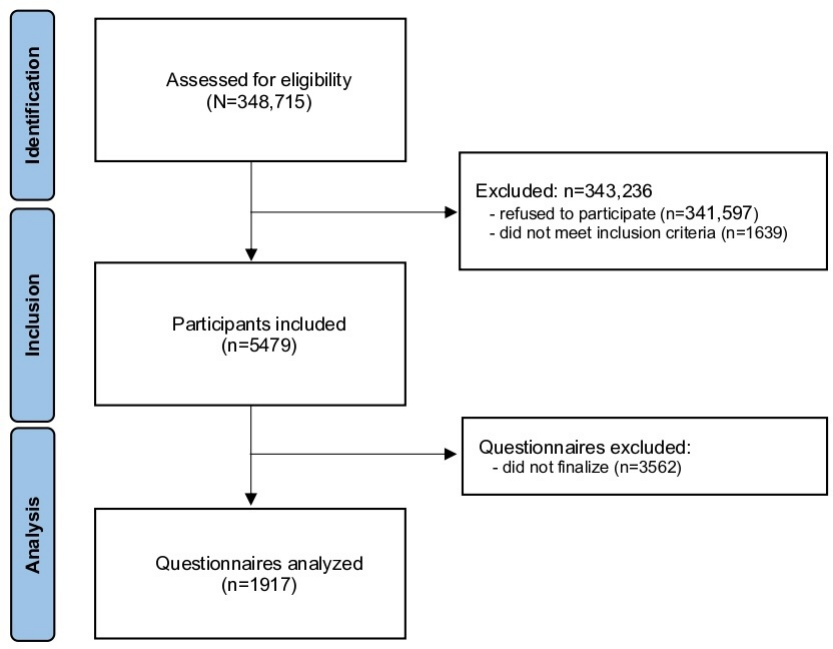
Flowchart of the study.

Figure 1 represents the flowchart of the study. A total of 348,715 persons were assessed for eligibility, among whom 343,236 were excluded (n=1639 did not meet the inclusion criteria and n=341,597 refused to participate). Finally, 5479 persons were included and 1917 completed the 3 questionnaires.

Table 1 presents the sociodemographic characteristics and behavioral attitudes to OH management of the participants. Participants had a mean age of 45.6 (SD 14.7) years. Among participants, 54.2% were women (n=1038), 70.9% were in a relationship (n=1359), and 52.8% were retired (n=1012). For education, 47.4% of participants had a level of 3 (n=909). Most of the participants reported visiting a dentist annually (n=1336, 69.7%), within the last 6 months (n=888, 46.3%), and for a simple check-up (n=1508, 78.7%).

**Table 1.** Sociodemographic characteristics and behavioral attitudes to oral health management of the participants (n=1917).

Variables		Participants, n (%)
**Age range (years)**		
	18‐29	21 (1.1)
	30‐39	74 (3.9)
	40‐49	249 (13)
	50‐59	432 (22.5)
	60‐69	637 (33.2)
	70‐79	437 (22.8)
	≥80	57 (3)
	MD^a^	10 (0.5)
**Gender**		
	Male	879 (45.9)
	Female	1038 (54.1)
**Education level**		
	Level 1	241 (12.6)
	Level 2	766 (39.9)
	Level 3	909 (47.4)
	MD	1 (0.1)
**Professional situation**		
	Active	845 (44.1)
	Retired	1012 (52.8)
	MD	60 (3.1)
**Professional category**		
	A	990 (51.7)
	B	681 (35.5)
	C	246 (12.8)
**Family situation**		
	Single	556 (29)
	In relationship	1359 (70.9)
	MD	2 (0.1)
**Have children**		
	Yes	1517 (79.1)
	No	400 (20.9)
**Children living in the household**		
	Yes	503 (26.2)
	No	1414 (73.8)
**Chronic disease**		
	Yes	703 (36.7)
	No	1207 (63)
	MD	7 (0.3)
**Frequency of dental visits**		
	≤1 year	1336 (69.7)
	Every 2 to 3 years	401 (20.9)
	Never or only in case of pain	180 (9.4)
**Last visit to the dentist**		
	<6 months	888 (46.3)
	6 months-1 year	567 (29.6)
	>1 year	458 (23.9)
	MD	5 (0.2)
**Reason for the last visit to the dentist**		
	Control	1508 (78.7)
	Emergency	385 (20.1)
	MD	24 (1.2)

a MD: missing data.

### OHL, OH Knowledge, and General HL of All Participants

Table 2 presents descriptive statistics for the OHLI-F, OH knowledge questionnaire, and s-TOFHLA.

OHLI-F scores ranged from 15.8 to 97.4, with a mean of 80.9 (SD 7.9). An adequate OHLI-F score was obtained by 84% of participants (n=1610). For the reading comprehension part, the participants obtained a mean score of 43.4 (SD 3.6), while for the numeracy part, the mean score was 37.5 (SD 6.0). The OH knowledge score ranged from 82.4 to 94.1, with a mean 87.6 (SD 7.9); the score was adequate for 90.6% of participants (n=1736). Consequently, 9.5% (n=181) and 16% (n=307) of participants had inadequate or marginal OH knowledge and OHLI-F scores, respectively. The scores for s-TOFHLA were higher than for OHLI-F, and s-TOFHLA scores ranged from 97.2 to 100.0, with a mean of 98.2 (SD 2.8). An adequate s-TOFHLA score was obtained by 99.9% of participants (n=1915).

The OHLI-F reading comprehension score was significantly higher than the OHLI-F numeracy score (*P*<.001 with a Welch 2-sample *t* test and paired *t* test).

**Table 2.** Descriptive analysis of Oral Health Literacy Instrument, French version (OHLI-F), oral health (OH) knowledge questionnaire, and Short Test of Functional Health Literacy in Adults (s-TOFHLA) (n=1917).

		OHLI-F			OH knowledge^c^	s-TOFHLA^c^
		OHLI-F reading comprehension^a^	OHLI-F numeracy^a^	OHLI-F summary^b^^c^		
**Score**						
	Mean (SD)	43.4 (3.6)	37.5 (6.0)	80.9 (7.9)	87.6 (10.5)	98.2 (2.8)
	95% CI	43.2‐43.6	37.2‐37.8	80.6‐81.3	87.1‐88.1	98.1‐98.4
	Range	10.5‐50.0	0.0‐50.0	15.8‐97.4	5.9‐100.0	61.1‐100.0
**Score level (participants), n (%)**						
	Inadequate (0‐59)	—^d^	—	38 (2)	58 (3.1)	0 (0)
	Marginal (60-74)	—	—	269 (14)	123 (6.4)	2 (0.1)
	Adequate (75-100)	—	—	1610 (84)	1736 (90.6)	1915 (99.9)

a Score out of 50.

b This score is the sum of OHLI-F reading comprehension and OHLI-F numeracy.

c Score out of 100.

d Not applicable.

### Determinants of Literacy Levels Among Civil Servants in France

The variance analysis of OHLI-F is presented in Table 3. Among the sociological variables analyzed, age, education level, and professional category significantly impacted OHLI-F, OH knowledge, and s-TOFHLA levels. The score for OH knowledge was also significantly impacted by gender.

**Figure 2.**
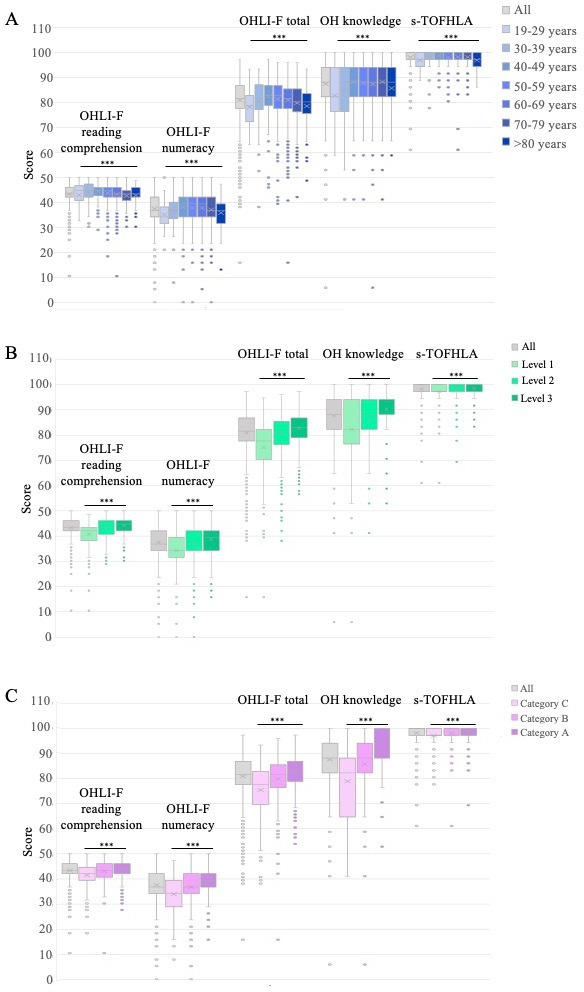
Oral Health Literacy Instrument, French version (OHLI-F), oral health (OH) knowledge questionnaire, and Short Test of Functional Health Literacy in Adults (s-TOFHLA) scores according to (A) age (years), (B) education level (1 to 3), and (C) professional category (categories A to C). ***P<.001.

The levels of OHL, OH knowledge, and general HL according to age, education level, and professional category are presented in Figure 2. Scores increased between ages 19 and 49, then decreased after age 49. They increased with the level of education and with the professional category.

**Table 3.** Variance analysis of sociological variables and scores on the Oral Health Literacy Instrument, French version (OHLI-F), oral health (OH) knowledge questionnaire, and Short Test of Functional Health Literacy in Adults (s-TOFHLA).

	OHLI-F (*P* value)	OH knowledge (*P* value)	s-TOFHLA (*P* value)
Age	<.001	.01	<.001
Gender	.07	<.001	.68
Education level	<.001	<.001	<.001
Professional situation	0.27	.24	.57
Professional category	<.001	<.001	<.001
Family situation	.17	.17	.60
Having children	.67	.49	.18
Children living in the household	.45	.36	.52
Chronic disease	.77	.79	.85
Frequency of dental visits	.50	.26	.63
Last visit to the dentist	.61	.57	.40
Reason for the last visit to the dentist	.10	.13	.06

### Determinants of Adequate OHL, OH Knowledge, and HL Levels Among Civil Servants in France

#### Determinants of an Adequate OHL Level

**Figure 3.**
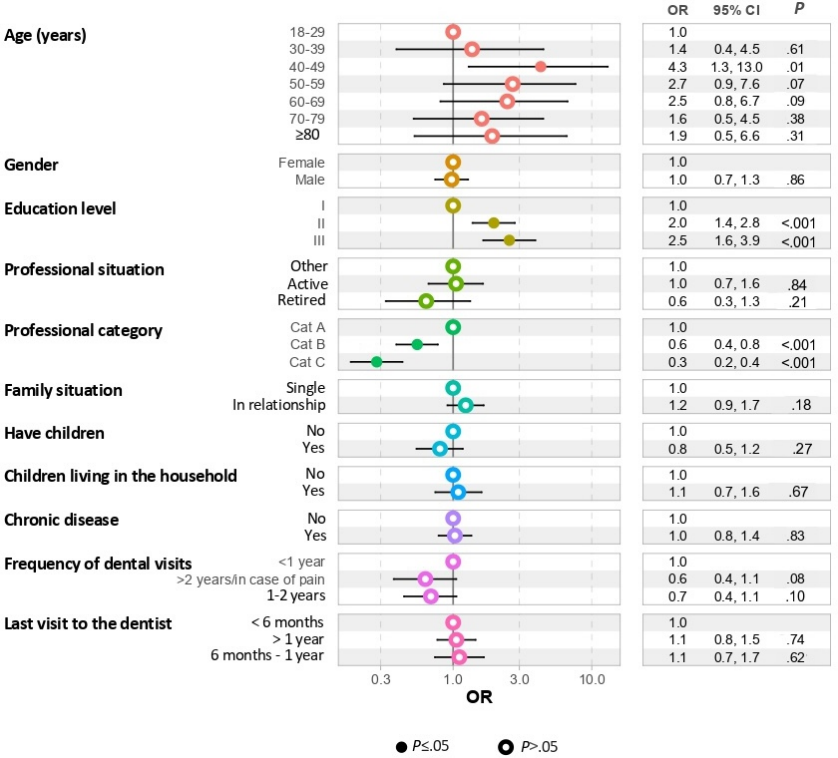
Binary logistic regression performed to evaluate the determinants of Oral Health Literacy Instrument, French version (OHLI-F) level. Education level: level 1 (low; <baccalaureate), level 2 (moderate; from baccalaureate to baccalaureate plus 2 years), and level 3 (high; >baccalaureate plus 2 years). Professional category (cat): cat A (hierarchically superior conceptual, managerial, and supervisory grades and positions, as well as teaching positions), cat B (middle management, application, and editorial positions), and cat C (executive positions). OR: odds ratio.

The results of the binary logistic regressions performed to evaluate the determinants of the OHLI-F are presented in Figure 3. A low OHLI-F score was significantly associated with a low professional category for B (odds ratio [OR] 0.6, 95% CI 0.4-0.8; *P*<.001) or C (OR 0.3, 95% CI 0.2-0.4; *P*<.001). A high OHLI-F score was significantly associated with age ranging between 40 and 49 years (OR 4.3, 95% CI 1.3-13.9; *P*=.01) and with education level 2 (OR 2.0, 95% CI 1.4-2.8; *P*<.001) or level 3 (OR 2.6, 95% CI 1.6-3.9; *P*<.001).

#### Determinants of an Adequate OH Knowledge Level

**Figure 4.**
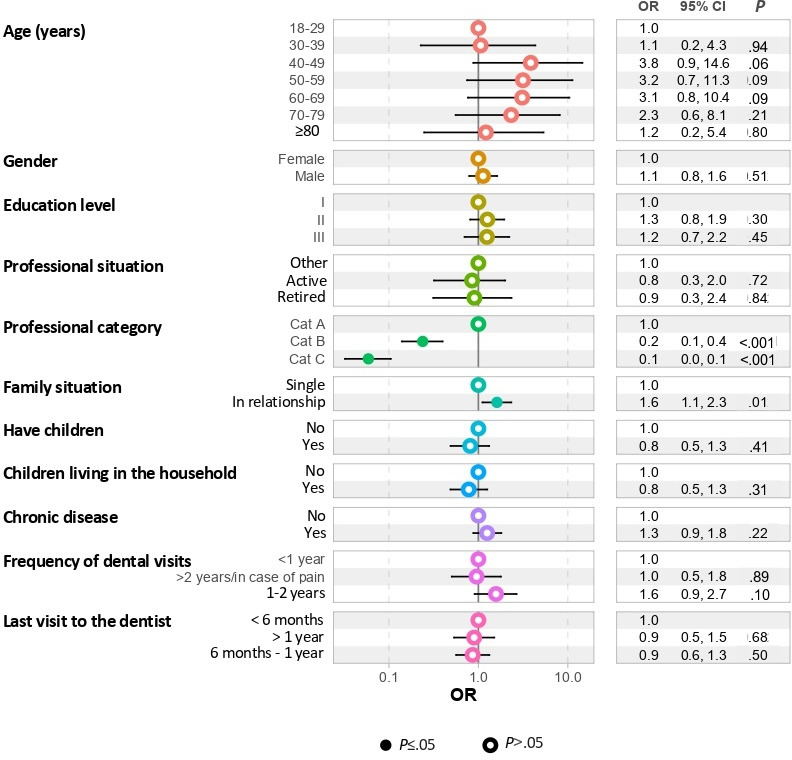
Binary logistic regression performed to evaluate the determinants of oral health knowledge. Education level: level 1 (low; <baccalaureate), level 2 (moderate; from baccalaureate to baccalaureate plus 2 years), and level 3 (high; >baccalaureate plus 2 years). Professional category (cat): cat A (hierarchically superior conceptual, managerial, and supervisory grades and positions, as well as teaching positions), and cat B (middle management, application, and editorial positions), and cat C (executive positions). OR: odds ratio.

The results of the binary logistic regressions performed to evaluate the determinants of an adequate OH knowledge level are presented in Figure 4. A low OH knowledge score was significantly associated with a low professional category for B (OR 0.2, 95% CI 0.1-0.4; *P*<.001) or C (OR 0.1, 95% CI 0.0-0.1; *P*<.001). Persons in a relationship had a significantly higher OH knowledge score (OR 1.6, 95% CI 1.1-2.3; *P*=.01). A high OH knowledge score was associated with an age between 40 and 79 years (OR 2.3, 95% CI 0.6-8.1; *P*=.21), but after 80 years (OR 1.2, 95% CI 0.2-5.4; *P*=.80), the OH knowledge score decreased and was similar to that of those aged between 18 and 39 years (OR 1.1, 95% CI 0.2-4.3; *P*=.94).

#### Determinants of an Adequate General HL Level

Given that all except 2 participants had adequate levels of general HL, the results of the logistic regression were not significant, and no determinants were identified.

### Analysis of the Correlation Between OHL, OH Knowledge, and General HL

Table 4 presents the matrix of correlations between OHLI-F, OHLI knowledge, and s-TOFHLA. OHLI-F was highly correlated with OH knowledge, but OHLI-F and OH knowledge had a low correlation with s-TOFHLA.

**Table 4.** Matrix of correlations between Oral Health Literacy Instrument, French version (OHLI-F), oral health (OH) knowledge questionnaire, and Short Test of Functional Health Literacy in Adults (s-TOFHLA).

		OHLI-F	OH knowledge	s-TOFHLA
**OHLI-F**				
	*r*	1	0.70	0.43
	*P* value	—^a^	<.001	<.001
**OH knowledge**				
	*r*	0.70	1	0.43
	*P* value	<.001	—	<.001
**s-TOFHLA**				
	*r*	0.43	0.43	1
	*P* value	<.001	<.001	—

a Not applicable.

## Discussion

### Principal Results

The WHO has emphasized the need to combat the burden of oral diseases and to include oral diseases in initiatives to prevent NCDs [1]. Improving OH and reducing OH inequalities is linked to the level of OHL [35]. It is therefore essential to know the level of OHL in order to implement appropriate preventive measures [36,37]. In order to reach a large number of people, these preventive actions must be deployed in the workplace. In France, civil servants represent several socioprofessional levels with different levels of education; they therefore represent an excellent study model with results that are transferable to the general population as a whole.

To our knowledge, this study is one of the largest studies in terms of number of participants analyzing OHL and general HL levels [31,38]. In addition, it is the first to focus on civil servants in France. Thus, this study represents an antecedent to the implemention of efficient preventive actions in the workplace. The population studied had a number of strengths that allow the results to be applied to the general population. First, this population included people of all ages (18 years to more than 80 years), marital status, and levels of education. Second, the public service population covers the majority of socioprofessional categories, with a wide variety of public occupations (eg, clerks, customs officers, librarians, police officers, state architects, magistrates, port officers, and prison staff). Third, this population is composed of different level of socioprofessional categories; the 3 categories (A, B, and C) cover jobs with different responsibilities, qualifications, remuneration, and recruitment conditions [39,40]. In this study, 51.7% (n=990) of participants belonged to category A, 35.5% (n=681) to category B, and 12.8% (n=246) to category C, which is in line with the distribution of civil servants in France who belong to category A (56%), B (24%), and C (21%) [24]. Fourth, this population has access to workplace prevention initiatives even after retirement.

Our results indicated that civil servants had an OHLI-F mean score of 80.9 (SD 7.9) and an OH knowledge mean score of 87.6 (SD 10.5). The majority had an adequate level of OHL (n=1610, 84%), OH knowledge (n=1736, 90.6%), and HL (n=1915, 99.9%). Consequently, 9.5% (n=181) and 16% (n=387) of participants had inadequate or marginal OH knowledge and OHLI-F scores, but none had an inadequate level of general HL. A low correlation between HL and OHL was observed. French civil servants had a higher level of HL than OHL and had a significantly higher level of OHLI-F reading comprehension (mean 43.4, SD 3.6) than OHLI-F numeracy (37.5, SD 6.0).

Age, education level, and professional category were the 3 determinants of the level of OHL, OH knowledge, and HL. Levels increased with age up to 49 years, with education level, and with professional category (C < B < A). Conversely, the professional situation (active or retired) did not represent a determinant of the level of OHL, OH knowledge, and HL. Working people obtained nonsignificantly higher OHLI scores than retirees, while no difference was observed between the 2 groups for the OH knowledge score.

More particularly, the determinants of an adequate level of OHLI were age, education level, and occupational category. For OH knowledge, persons in a relationship had a significantly higher score, and persons with a low professional category (B or C) had a less-adequate score.

### Comparison With Prior Work

Regarding the level of OHL and OH knowledge, civil servants had a higher level than observed in the French population, obtaining an OHLI mean score of 73.5 (SD 13.6) and a mean OH knowledge score of 79.8 (SD 15.3) [30]. This difference could be due to the fact that the sample size was smaller in the study by Clément et al [30]. Compared with other countries, OHLI scores are higher in Canada (87.2, SD 10.2) [28] and the United States (81.6, SD 13.6) [41] but lower in Russia (77.2, SD 14.5) [42], Malaysia (75.1, SD 15.6) [29], and Chile (37.1, SD 9.0) [43]. The low score observed in Chile may be due to the fact that the Spanish OHLI questionnaire used was not well adapted, because there are phonological, grammatical, and lexical variations in Spanish in Hispanic America [43]. The OH knowledge score observed in the French civil servants was higher than in the Russian population (63.8, SD 16.9), American population (60.8, SD 3.1), Canadian population (57.0, SD 26.1), and Chilean population (10.0, SD 3.6).

Regarding the reading comprehension and numeracy scores, in all countries that measured OHLI with this scale, the scores were similar [28,30,41-43], except for France [30]. Indeed, in France, for the population as a whole, the reading comprehension score (41.4, SD 6.4) was higher than the numeracy score (34.2, SD 8.5) [30], as observed in the population of civil servants in this study. Previous studies have shown that low numeracy reduces adherence to medication, skews perceptions of the benefits and risks of screening, hinders access to treatment, undermines risk communication (restricting prevention initiatives for the most vulnerable), and seems to have a negative effect on medical outcomes [44]. Low numeracy is also associated with higher sensitivity to external factors that do not affect objective numerical data but are linked to biases in judgment and decision-making (eg, the influence of framing and ratio bias effects) [45]. Thus, in France, health promotion actions should consider this specifically and should target the improvement of numeracy. For this, prevention initiatives targeting people with low numeracy should be implemented with the aim of improving their understanding of digital information. These actions would benefit all patients, even those with high numeracy [46].

Regarding the determinants of the level of OHL and HL, the education level was also underlined in the studies of Clément et al [30], Ramlay et al [29], McCarlie et al [41], and Blizniuk et al [42]. In the study of Sabbahi et al [28], the education level did not have a significant impact. To our knowledge, for the OHLI questionnaire, the link between professional category or professional situation and OHL or HL have not been studied. However, this study demonstrated that these professional factors are major determinants of OHL and HL. Ehmann et al [47] demonstrated a bidirectional relation between work and HL. Work is a determinant of HL and the benefits of adequate HL have an impact on all life activities, including work. In addition, age appears to be a determinant of OHL, as previously described [48,49]. Although there is considerable individual variability, age-related decline in literacy is linked to a reduction in comprehension and fluency of reasoning as a result of physiological aging.

Regarding the correlation between OHLI and s-TOFHLA, a weak correlation was observed, in contrast to previous studies that demonstrated strong correlations between the Malay version of the OHLI and s-TOFHLA [29] and between the OHLI and TOFHLA [28]. This could be explained by the fact that s-TOFHLA shows errors in identifying people with low HL in specific dimensions of HL, such as numeracy, which was the case in our sample. The correlation between OHLI and s-TOFHLA therefore needs to be qualified, and efforts are needed to develop more-encompassing and practical strategies for identifying those with low HL for use in research and clinical practice [50]. Therefore, in order to assess the correlation between OHL and HL, it might be interesting to use a questionnaire that assesses HL on the basis of reading comprehension and numeracy, such as TOFHLA [51].

### Limitations

This study had several limitations. First, the s-TOFHLA was chosen to assess literacy levels because it is validated in French, easy to use, and short [33], but this questionnaire only includes a reading comprehension section and not a numeracy section. The TOFHLA [51], the long version of the s-TOFHLA, could have been chosen as it includes both sections, like the OHLI, and was used as a model for the development of the OHLI. However, it is not available in French and takes 22 minutes to complete. Thus, completing the OHLI, OH knowledge, and HL questionnaires would have led to participant exhaustion and thus bias in the assessment. Moreover, the other HL questionnaires available in French (the Functional, Communicative and Critical Health Literacy scale [52], the Health Literacy Questionnaire [53], the European Health Literacy Survey Questionnaire [54], and the Single-Item Literacy Screener [55]) are not based on reading comprehension and numeracy.

Second, the OHLI-F was used to assess the OHL level because it is the only questionnaire validated in French. Other OHL questionnaires containing reading comprehension and numeracy sections could have been used, such as the Comprehensive Oral Health Literacy Scale [56] or the Test of Functional Health Literacy in Dentistry [57].

Third, even if our results seem generalizable to the entire public service, this study only included state agents. This profession remains the most important among the 5.67 million French civil servants, divided into 3 parts (state, territorial, and hospital), including 2.52 million for the state public service, that is, 1.46 million people after excluding the national education department [24].

Fourth, as the aim of this study was to determine the level of literacy required to implement OH promotion programs in the workplace, the composition of our population (n=1190, 59% were aged over 60 years and n=1012, 52.8% were retired) could represent a bias. However, they are a target population because aging affects their literacy, OH, and general health levels. In France, a particularity is that retirees can access workplace programs through their health insurance.

Finally, it is possible that our results are related to selection bias, as some of the nonrespondents may have refused to answer the HL questionnaires to hide their low level of HL, thus underestimating the prevalence of low HL [58].

### Perspectives

The purpose of this research was to investigate the level of HL and, more specifically, OHL among civil servants in France in order to contribute to the formulation of prevention strategies. These strategies will target increasing the knowledge of OHL in this professional category of the state civil service, which includes more than 5 million active and retired civil servants [24,59]. These prevention strategies should be oriented toward a community-based learning model focusing on the most vulnerable risk groups. For this, the workplace appears to be an opportunity [20,21,60]. Even if most civil servants had an adequate level of OHL and global HL, 2 high-risk groups have been identified for the implementation of a public health literacy program. First, the category C agents have been determined to be the most vulnerable socioprofessional category, yet these employees constitute the vast majority of territorial civil servants (75.6%), half of hospital civil servants (48%), and only 20% of state civil servants. Second, retired people are a particularly vulnerable group, with low levels of HL. The average retirement age for civil servants (excluding military personnel) is 62 years. This group represents 2.5 million people in the state civil service, and is composed only of people older than 62 years [59].

As the Report of the French National Health Commission advocates [61], our results suggest the implementation of an ambitious action program, with a particular focus on category C civil servants and retired civil servants. Our main recommendations focus on three areas: (1) create a favorable environment for the development of HL, which requires advocacy with both national and local decision-makers; (2) mobilize relevant resources and mechanisms to develop HL among vulnerable civil servants; and (3) develop, evaluate, investigate, and disseminate best practices specific to the at-risk groups, which to date require further clarification and evaluation. Preventive measures should be targeted primarily at category C civil servants and retired civil servants. For example, activities such as “teach-back,” which has a real role to play in literacy, could help to improve OHL [62]. In addition, tools could be proposed such as (1) modern technologies to improve the learning process, (2) face-to-face or online meetings and courses for empowerment and health promotion, (3) freely accessible electronic books or journals for information, and (4) specific applications targeting HL teaching [63].

### Conclusions

The civil service represents 19.8% of total jobs in France, to which it is possible to add the 5.5 million retirees who are part of the same administrative system. Targeting this population to raise awareness and promote OHL, which has a considerable impact on health outcomes, is therefore an important public health issue. The workplace is at the heart of the health promotion strategy, with a focus on vulnerable groups such as category C professionals and older or retired civil servants. Future investigations will need to detect and investigate appropriate preventive communication procedures adapted to the literacy level of professional categories of workers and focused on numeracy skills. This should make it possible to achieve greater disease equity and combat the burden of NCDs.

## Supplementary material

10.2196/58942 Multimedia Appendix 1 STROBE (Strengthening the Reporting of Observational Studies in Epidemiology) checklist.

## Abbreviations

COHL: Comprehensive Oral Health Literacy
HL: health literacy
NCD: noncommunicable disease
OH: oral health
OHL: oral health literacy
OHLI: Oral Health Literacy Instrument
OHLI-F: Oral Health Literacy Instrument, French version
OR: odds ratio
s-TOFHLA: Short Test of Functional Health Literacy in Adults
STROBE: Strengthening the Reporting of Observational Studies in Epidemiology
WHO: World Health Organization
